# Echocardiography in shock management

**DOI:** 10.1186/s13054-016-1401-7

**Published:** 2016-08-20

**Authors:** Anthony S. McLean

**Affiliations:** Nepean Hospital, PO Box 63 Penrith, Sydney, NSW 2751 Australia

**Keywords:** Critical care echocardiography, Shock assessment, Hemodynamic echo evaluation

## Abstract

Echocardiography is pivotal in the diagnosis and management of the shocked patient. Important characteristics in the setting of shock are that it is non-invasive and can be rapidly applied.

In the acute situation a basic study often yields immediate results allowing for the initiation of therapy, while a follow-up advanced study brings the advantage of further refining the diagnosis and providing an in-depth hemodynamic assessment. Competency in basic critical care echocardiography is now regarded as a mandatory part of critical care training with clear guidelines available. The majority of pathologies found in shocked patients are readily identified using basic level 2D and M-mode echocardiography. A more comprehensive diagnosis can be achieved with advanced levels of competency, for which practice guidelines are also now available. Hemodynamic evaluation and ongoing monitoring are possible with advanced levels of competency, which includes the use of colour Doppler, spectral Doppler, and tissue Doppler imaging and occasionally the use of more recent technological advances such as 3D or speckled tracking.

The four core types of shock—cardiogenic, hypovolemic, obstructive, and vasoplegic—can readily be identified by echocardiography. Even within each of the main headings contained in the shock classification, a variety of pathologies may be the cause and echocardiography will differentiate which of these is responsible. Increasingly, as a result of more complex and elderly patients, the shock may be multifactorial, such as a combination of cardiogenic and septic shock or hypovolemia and ventricular outflow obstruction.

The diagnostic benefit of echocardiography in the shocked patient is obvious. The increasing prevalence of critical care physicians experienced in advanced techniques means echocardiography often supplants the need for more invasive hemodynamic assessment and monitoring in shock.

## Background

Whether the cause of shock is unknown, suspected, or established, echocardiography is utilized in its diagnosis and management and to monitor progress. It is recommended as the modality of first choice in consensus guidelines [1]. No other investigative bedside tool can offer a similar diagnostic capability, allowing for exact targeting of the underlying cardiac and hemodynamic problems whether it be the right heart, left heart, fluid perturbations, pericardial, or a cardiac response to vasoplegia as found in septic shock. The clinician needs to undertake a careful, structured echocardiographic examination, even in an emergency situation where urgency demands a rapid assessment.

Competency standards are well established for both basic and advanced critical care echocardiography and the scope of this review will cover both [2, 3] (Table 1). Both transthoracic echocardiography (TTE) and transesophageal echocardiography (TEE) expertise should be available, the latter being seen as part of the armamentaria of the advanced practitioner. It may be an iterative process whereby a basic assessment or rapid cardiac assessment by echo (RACE) is performed immediately in the deteriorating patient, with subsequent initiation of treatment, followed at a later time by a more detailed advanced echocardiographic assessment.

**Table 1:** Basic and advanced echocardiograph evaluation in the shocked patient

	Race	Advanced
Modality	2D, M-mode	2D, M-mode
		Colour Doppler
		Special Doppler
		TDI
Assessments	LV contraction	LV systolic function
	RV contraction	Diastolic function
	Intravascular fluid status	RV systolic function
	Pericardial tamponade	Intravascular fluid status
		Valve structure/function
		Pericardial tamponade
		Hemodynamics
		Pulmonary artery pressure
		Left atrial pressure
		Cardiac output
		Ventricular outflow obstruction

*LV* left ventricle, *RACE* rapid assessment by cardiac echo, *RV* left ventricle, *TDI* tissue doppler imaging

## Shock: definition and classification

Shock can be defined as a life‐threatening, generalized form of circulatory failure associated with inadequate oxygen delivery to the cells [1]. The four major underlying mechanisms, either alone or in combination, include inadequate circulating volume (cardiogenic shock), failure of pump function (hypovolemic shock), obstruction to blood flow (obstructive shock), and loss of vascular tone (vasoplegic shock). The diagnosis of acute circulatory failure includes the clinical signs of hypotension (not always present), poor peripheral perfusion determined by skin changes, especially cold, clammy, discolored skin, decreased urine output (<0.5 ml/kg/min), and altered mental function, including obtundation and confusion. It should be noted that a defined level of blood pressure to denote the presence of shock is not recommended [1, 4].

## Cardiogenic shock

Cardiogenic shock is the extreme end of the acute deteriorating heart failure spectrum. A global study involving 666 hospitals involving nearly 5000 patients admitted to hospital with acute heart failure found 36 % were first time episodes, 37 % were in pulmonary edema, and 12 % were in cardiogenic shock [5]. Overall hospital mortality was 12 %, rising to 18 % in those patients admitted to ICU. Cardiogenic shock carries the worst prognosis with improvements in mortality, from 70 to 50 %, resulting mainly from early revascularization. Consensus documents from major societies are available [6].

Although most of the literature pertaining to cardiogenic shock relates to underlying coronary artery pathology, the critical care physician encounters a broader range of pathologies, including sepsis, resulting in severe cardiac failure and echocardiography is the only bedside tool that can accurately elucidate the underlying pathology.

A RACE assessment, using only two-dimensional (2D) and M‐mode echocardiography demonstrates major underlying abnormalities rapidly in the acute scenario [7]. Overall left ventricular contraction, including ejection fraction, segmental wall motion abnormalities, right heart failure, clues to intravascular volume status, and pericardial tamponade, can be identified. However, advanced techniques involving the use of spectral Doppler and tissue Doppler imaging (TDI) yield much more information, providing both diagnostic and hemodynamic evaluation.

### Overall cardiac performance

The estimation of cardiac output by echocardiography (echo) is well validated. Although it can be measured using the 2D Simpson’s multidisc method, the use of pulsed-wave Doppler across the left ventricular output tract (LVOT) is more accurate [8]. This often supplants invasively acquired CO measurements unless continuous monitoring is considered important. Other parameters of overall cardiac function, such as myocardial performance index (MPI) and mitral annulus plane systolic excursion (MAPSE) are not well validated in critically ill subjects. The LVOT velocity time integral (VTI) as a single measure that can be used as a surrogate for the stroke volume with a normal value >20 cm [9]. A value above 18 cm implies an adequate stroke volume.

### Left ventricular systolic function

**Fig. 1:**
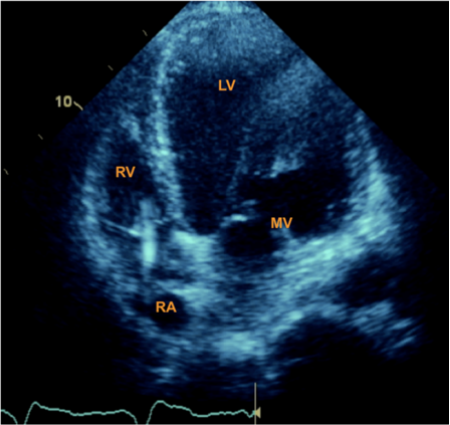
Grossly dilated left ventricle with biventricular pacing wire present in right heart in the apical four-chamber view. *LV* left ventricle, *MV* mitral valve, *RA* right atrium, *RV* right ventricle

Contractility is the ability of the myocardium to contract against a specific load for any given preload. Echo is used to measure contraction, which is measured as the degree of myocardial fiber shortening that occurs during systole. The most common cause of cardiogenic shock results from marked reduction in left ventricular contraction. The size of both the left atrium and ventricle may provide clues to the duration of the contractile impairment, with dilatation indicating a degree of chronicity (Fig. 1). Left ventricular ejection fraction (LVEF) is a traditional parameter which, though far from ideal, can be a helpful guide. The Simpson’s multidisc method can be applied in RACE. Subjective evaluation or “eyeballing” the LVEF is reasonably accurate with experience but objective measurement should always be considered in the advanced study. It is sufficiently robust to be used regularly in large studies in the chronic heart failure setting where it serves as a prognostic marker [10]. When the endocardial border is difficult to visualize, contrast echo may enhance accuracy [11].

Interpretation needs to take into account the effects of arterial blood pressure (afterload), inotropes, and vasopressors. For example, a struggling left ventricle may appear normal in the presence of inotropes. Other cardiac pathologies need to be taken into account as a normal or high LVEF may misled the clinician into believing there is good cardiac function although marked diastolic or valvular dysfunction is present.

Fractional area change (FAC) has been used to assess the left ventricle with reasonable accuracy in surgical patients undergoing TEE during cardiac surgery [12]. It can be measured from either the parasternal short axis view (PSAX) using TTE or the transgastric view with TEE short axis views, using the difference between the end diastolic and end systolic areas divided by the end diastolic area, with a normal range being 38–60 %. The reliability is less certain in hemodynamically unstable patients, in the presence of segmental wall motion defects or left bundle-branch block, or where right ventricular dysfunction exists, and as a result is used less commonly in the ICU setting compared with the operating theatre.

The advanced practitioner can use a number of Doppler and TDI parameters to more accurately quantify left ventricular dysfunction. When mitral regurgitation is present, dP/dt can be calculated, a normal value being >1200 mmHg/s and markedly abnormal values being <800 mmHg/s [13]. Using TDI, the myocardial systolic velocity S’, measured from an average of readings from multiple segments, correlates with LVEF. In a study involving four basal segments, a S’ >7.5 correlated with an LVEF >50 % with a sensitivity of 79 % and specificity of 88 % [14]. Using an average of six basal segments, Gulati and colleagues found an S’ >5.4 indicated an LVEF >50 % with sensitivity 88 % and specificity 97 % [15]. It should be noted that S’ decreases with age and does not differentiate active contraction from tethering effects.

Other techniques currently under investigation, although contributing to left ventricular contraction assessment in the stable outpatient population, have yet to prove beneficial in the critically ill. Strain rate imaging and speckle tracking using global longitudinal strain have been demonstrated to identify systolic dysfunction in patients with normal LVEF in oncology and heart failure patients [16, 17]. The value in critically ill patients is still uncertain [18].

Any assessment of left ventricular contractility needs to take the presence or absence of identifiable segmental wall motion abnormalities into account; if present, urgent revascularization should be considered to enhance prognosis.

### Valvular pathology

**Fig. 2:**
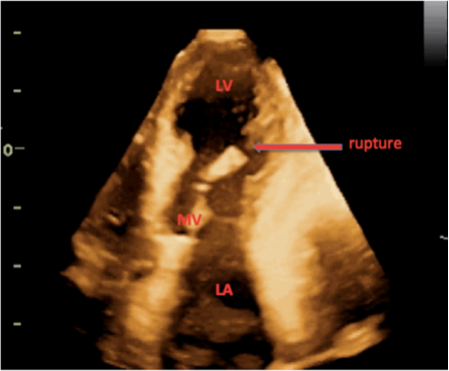
Ruptured mitral papillary muscle post infarction seen by 3D echocardiography from the apical four-chamber view view. *LA* left atrium, *LV* left ventricle, *MV* mitral valve

Echocardiographic investigation extends to possible valvular lesions, both acute and preexisting, like degenerative aortic stenosis and mitral regurgitation, frequently found in the older population. Acute lesions such as peri‐infarction rupture of a papillary muscle resulting in severe mitral regurgitation may necessitate urgent surgical repair of the valve. Initial examination of the valves in the acute setting, allowing for initiation of treatment, requires reasonable but not necessarily expert skills. A more comprehensive valve examination can be performed later by clinicians highly skilled in valve evaluation (Fig. 2).

### Left ventricular diastolic dysfunction

Approximately half the patients presenting with acute heart failure have preserved ejection fraction via a number of mechanisms, including diastolic dysfunction-reduced coronary flow reserve [19, 20]. High metabolic states frequently found in critically ill patients can exacerbate cardiac failure by worsening diastolic function. Although there is a comprehensive background to assessing left ventricular diastolic function, it was the advent of TDI that simplified the situation and brought analysis to the bedside. In particular, TDI analysis of the mitral annulus allows for rapid estimation of left atrial pressure (LAP), an important parameter in evaluating left ventricular function and preload.

The use of spectral Doppler of mitral inflow still remains paramount. Both an E/A ratio >2 and an E wave deceleration time <120 ms predict a LAP >20 mmHg [21]. With TDI, the mitral annulus e′ offers a quick guide to the presence of left ventricular diastolic dysfunction with a lateral e′ <10 and medial <7 cm/s highly suggestive of diastolic dysfunction and elevated left atrial pressures [22].

The E/e′ ratio, although still affected by loading conditions, is of considerable value by giving a guide to elevated left atrial pressures. The original description using patients with coronary disease or heart failure used an E/e′ <8 to indicate normal LAP and a value >15 gave an LAP >13 mmHg [23]. The average of the lateral and septal e′ measurements is recommended. Interestingly, recent international guidelines on assessing left ventricular diastolic dysfunction choose a discriminating average E/e′ value of 14 to identify elevated left atrial pressure [24].

The E/e′ value used to identify elevated left atrial pressures in patients on positive ventilation is less than that used in non-ventilated patients, around 12 using the average septal/lateral e′ rather than the classic 14–15 [25]. However, a precise and accurate value is unclear. Positive pressure ventilation affects left ventricular diastolic filling in a number of often opposing ways and the overall effects are difficult to predict. Increased intrathoracic pressure, by reducing systemic venous return, results in decreased left ventricular preload and, by decreasing the atrial–ventricular pressure gradient, reduces E and e′. Lung hyperinflation can decrease pulmonary vascular resistance when the volume increase is less than the functional reserve capacity but beyond this will increase the resistance with subsequent effects on right ventricular afterload and left ventricular preload. A lowering of the transmural pressure decreases afterload of the left-sided chambers, resulting in an increase in left atrial contractility and subsequent augmentation of ventricular filling, theoretically increasing A and a’, and even E and e′ [26]. In critically ill patients an E/e′ >13 is indicative of elevated left atrial pressure and, although very useful, is not without controversy [27, 28].

### Other considered pathologies in cardiogenic shock

The contribution of right heart function to shock will be covered in the “Hypovolemic shock” section. Post-infarction ventricular septal defects, although uncommon, often occur some days after the actual infarction and are usually catastrophic. The presence of new onset aortic regurgitation, particularly when it is associated with a pericardial effusion, should lead to investigation of dissection of the thoracic aorta. This requires a TEE.

## Hypovolemic shock

**Fig. 3:**
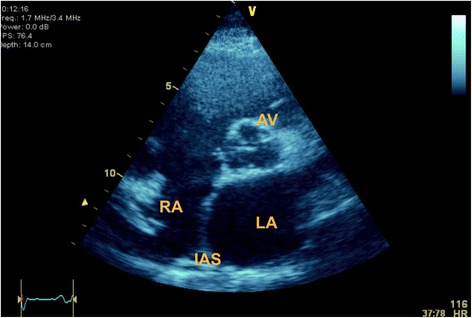
Bowing of interatrial septum from left to right indicating elevated left atrial pressure in PSAX view. *AV* aortic valve, *IAS* interatrial septum, *LA* left atrium, *RA* right atrium

Although particularly pertinent in suspected hypovolemic shock, assessment of intravascular volume is the starting point in all types of circulatory failure. Often clinically insufficient volume is readily evident but can be difficult to determine by physical examination alone. At the basic level of competency the clinician relies on 2D and M‐mode echocardiography only. When hypovolemia is severe, 2D views can be impelling when they show collapse of the left ventricular walls at end‐systole, the so‐called “kissing walls”. Conversely, fixed bowing of the atrial septum into the right atrium throughout the cardiac cycle implies elevated left atrial pressures and further fluid is not necessary (Fig. 3). It should be noted that neither of these signs are specific for intravascular fluid status. Left ventricular end diastolic area (LVEDA) appears to be helpful in assessing response to a volume load in anaesthetized patients undergoing surgery but unfortunately not in the critically ill patients [29].

Inferior vena cava (IVC) variation has been recognized as a useful parameter for some decades now and, while far from ideal, is a good place to start. Numerous studies have explored refining the technique using vessel diameter variation in response to the respiratory cycle, maximum diameter, and percentage of diameter alteration to assess right atrial pressure (RAP) [30].

Guidelines recommend that in the spontaneously breathing patient an IVC diameter (D) <21 mm that collapses with a sniff (i.e., the caval or collapsibility index [CI = (D_max_ − D_min_)/D_max_ × 100 %]) indicates a normal RAP of 3 mmHg, whereas an IVC diameter >21 mm that collapses <50 % with a sniff indicates a RAP of >15 mmHg [31]. In a study of 73 emergency patients over 50 years of age, Nagdev and colleagues demonstrated, without taking IVC diameter into account, that an IVC collapse of >50 % had a positive predictive value of 87 % and a negative predictive value of 96 % of a central venous pressure <8 mmHg with a receiver operating curve (ROC) of 0.93 [32]. In a study on IVC diameter variation following fluid administration to hypovolemic trauma patients, inadequate dilatation indicated insufficient circulating blood volume despite normalization of blood pressure [33].

The transition from the cardiology setting to critical care practice resulted in a conceptual change, with changes in IVC diameter being used to assess fluid responsiveness rather than pressure equivalents.

For practical purposes, in the acute setting for the spontaneously breathing patient in shock, the IVC diameter is measured within 0.5–3 cm from the caval–right atrial junction in the subcostal view and when the diameter is less than 10 mm the patient is likely to respond to fluid, but when greater than 20 mm that is unlikely. Collapse of >50 % between the diameters of 10–20 mm should result in a trial of fluid. In the patient on fully supported positive pressure ventilation, the distensibility index (dIVC) is a good guide to fluid responsiveness. The dIVC is calculated as the ratio of (D_max_ − D_min_)/D_min_, with a threshold of 18 % discriminating between responders and non-responders with 90 % sensitivity and 90 % specificity [34].

There are pitfalls when performing IVC measurements and the operator should take care to obtain a good longitudinal view with the scan plane parallel to the IVC and the probe tilted in both directions to obtain the largest diameter. As the IVC can move inferiorly during inspiration, two different segments of the vessel can be inadvertently measured using M‐mode, so 2D measurements, with the highest possible frame rate, are recommended. Neither collapse nor distension of the IVC during respiratory ventilation should be used on patients receiving partial ventilatory support and, even in both groups described above, the clinician can only occasionally confidently predict fluid responsiveness on the IVC alone. Furthermore, the presence of right heart failure, increased intra‐abdominal pressure, or pericardial fluid makes the use of IVC even less reliable.

When TEE is being applied, the superior vena cava in the fully supported ventilated patient can be used and a collapse of >36 % during inspiration discriminates fluid responders from non‐responders with a sensitivity of 90 % and specificity of 100 % [35].

The use of static measurements to assess fluid status is recognized to be inadequate in the majority of situations and dynamic techniques need to be applied. Administration of a bolus of intravenous fluid, passive leg raising, and positive pressure ventilation-induced variation in stroke volume (SV) and CO are commonly employed. As a guide, fluid responsiveness is determined if there is, on average, a >15 % increase in SV or CO. The underlying physiology is well covered elsewhere and the focus of this review is on the practical application of echocardiography in shocked patients. [36]. Essentially, large SV variations occur on the steep part of the Starling curve and small variations on the flat part of the curve and either the SV or a surrogate measure, such as the velocity time integral (VTI), can be measured echocardiographically in response to the maneuver chosen. Doppler application uses the relationship between the velocities of blood flowing across the LVOT at the level of the aortic valve annulus or, alternatively, flow across the right ventricular outflow tract (RVOT) at the level of the pulmonary valve annulus, combined with the cross-sectional area (CSA = *π*(LVOT diameter/2)^2^) of the chosen location. CO and SV are measured using pulsed‐wave Doppler with the sample volume placed at the level of the aortic annulus for left ventricular outflow (where SV = VTI × CSA and CO = SV × Heart rate). Care must be taken to properly align the Doppler beam to the flow and VTI is measured by tracing the modal velocity.

### Selected maneuvers

#### Intravenous fluid administration

Application of a bolus of intravenous fluid has long been used to assess fluid responsiveness with clinical parameters, especially systemic blood pressure, used as an endpoint. Pulse pressure variation is used as blood pressure does not always reflect fluid responsiveness, particularly when other factors, such as impaired left ventricular contraction or marked vasoplegia, exist. With increasing awareness of the perils of excessive fluid administration, the practice of mini‐boluses of fluid is attractive. This is particularly the case in patients with impaired left ventricular function who are at greater risk of acute pulmonary edema. In a study on 39 low volume-ventilated critically ill patients, sub-aortic VTI was measured following an initial 100 ml of starch administered over 1 minute followed by another 400 ml over 14 minutes. A change in VTI of >10 % after the first 100 ml predicted fluid responsiveness with a sensitivity and specificity of 95 % and 78 %, respectively (area under curve (AUC) = 0.92) [37].

#### Respiratory variation

During the inspiratory phase of positive pressure ventilation, right ventricular output is reduced because of a decrease in venous return (increased intrathoracic pressure) causing a subsequent decrease in left ventricular output after two to three beats if both ventricles are volume responsive. These approaches are limited to fully ventilated patients and studies were performed using tidal volumes of 8–10 ml/kg. As smaller tidal volumes are not proven to be diagnostically helpful, it may be necessary to temporarily increase these to 8 ml/kg. A SV variation >10 % is highly predictive of volume responsiveness [38]. An increase in a respiratory rate from 14-16 to 30-40 breaths per minute in hypovolaemic patients resulted in a decrease in pulse pressure variation from 21 % to 4 % and in respiratory variation in aortic flow from 23 % to 6 %, with no accompanying change in cardiac index [39].

One factor to consider when using positive pressure ventilation to predict fluid responsiveness in mechanically ventilated patients is right ventricular function. Using TDI of the tricuspid annulus, Mahjoub and colleagues [40] found that an S’ <15 cm/s yields a false positive positive pressure ventilation result.

#### Passive leg raising

Passive leg raising (PLR) has been demonstrated to be applicable in both spontaneously breathing and ventilated patients. Correct positioning of the patient is essential. CO is measured using pulsed‐wave Doppler. An increase in CO or SV of >12 % during PLR was highly predictive of fluid responsiveness with an AUC of 0.89 for the cardiac index and 0.9 for the SV. Sensitivity and specificity values were 63 and 89 % for CO, and 69 and 89 % for SV, respectively [41]. Using esophageal Doppler, Monnet and colleagues [42] demonstrated in 37 ventilated patients that a PLR increase of >10 % aortic blood flow predicted fluid responsiveness with a sensitivity of 97 % and specificity of 94 %. A false positive response to PLR may occur in the presence of increased intra-abdominal pressure.

Assessing intravascular volume should be the first step in managing all types of shock. A basic approach using RACE generally identifies gross hypovolemia. Where uncertainty exists about intravascular fluid status, more advanced techniques utilizing Doppler and dynamic maneuvers should be employed.

## Obstructive shock

The common mechanism in patients with obstructive shock is resistance to blood flow through the cardiopulmonary circulation. Specific pathological diagnoses are acute pulmonary embolus, cardiac tamponade, and dynamic outflow obstruction; on occasion, it also occurs as a result of a type A dissection of the thoracic aorta or a tension pneumothorax. Constrictive pericarditis is a rare cause of obstructive shock.

### Acute pulmonary embolus

Classic right heart changes identified by echo are diagnostically and prognostically very helpful, indeed essential, in the shocked patient [43]. Diagnostic criteria include dilated right heart chambers, changes in right ventricular contraction, elevated pulmonary artery pressures, decreased cardiac output, and intra‐cavity emboli. Dilatation of the right ventricle is readily assessed in the apical four-chamber view with a right ventricle/left ventricle area ratio >0.6; gross dilatation is seen with a ratio >1.0. [44]. Right atrial area/volume is best measured by the Simpson’s method in the apical four-chamber view. Right ventricular contraction can be normal, hyperdynamic soon after the insult of the pulmonary embolus, or hypodynamic in the later stages. Tricuspid annulus plane systolic excursion (TAPSE) is a reasonably reliable and easily obtainable parameter for overall right ventricular contraction with a normal value being >16 mm. TDI, using the lateral tricuspid annulus S’ velocity, is a useful tool to identify early right ventricular dysfunction. A right ventricular S’ velocity <11.5 cm/s predicts right ventricular dysfunction (right ventricular ejection fraction <45 %) with a sensitivity of 90 % and specificity of 85 % [45]. In regular daily practice an S’ of 10 cm/s is a useful and easily remembered number to differentiate between normal and abnormal right ventricular systolic function.

The McConnell sign, where good apical but poor free wall contraction is seen, is considered an important sign by some [46]. However, it is also found in right ventricular infarction and its specificity for pulmonary embolism has been called into question [47, 48]. The pulmonary artery systolic pressure is most commonly obtained by converting the peak velocity of the tricuspid regurgitation to pressure using the modified Bernoulli equation and adding to the right atrial pressure. Care needs to be taken to obtain accurate Doppler signals. In the absence of a reliable tricuspid regurgitant signal, the acceleration time of the pulmonary ejection signal (PAcT) is used [49].

**Fig. 4:**
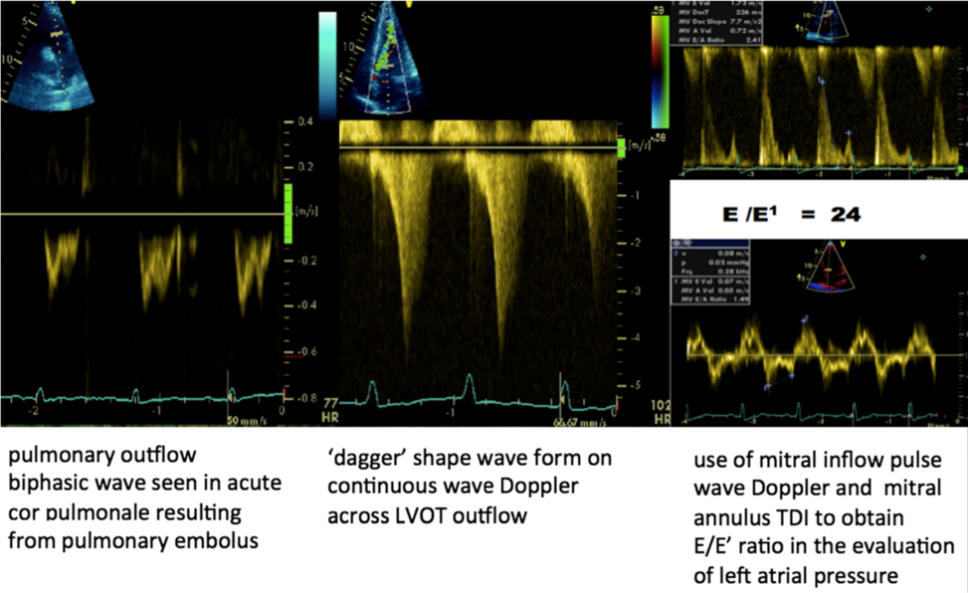
Examples of assessing the shocked patient using spectral Doppler

As a guide, a PAcT of 70–90 ms indicates a pulmonary artery systolic pressure of >70 mmHg. The presence of mid‐systolic notch also indicates severe pulmonary hypertension (Fig. 4).

The classic 2D sign of pulmonary hypertension resulting in a marked increase in right ventricular pressures is paradoxical septal motion, whereby a D‐shaped left ventricle is seen on the parasternal short axis view. The presence of mobile thrombo‐emboli in the right heart chambers, inferior vena cava, or pulmonary artery are occasionally seen and may push the clinician to early administration of thrombolytic therapy. Examination of the left ventricle is also informative in severe acute pulmonary embolus, with small chamber size and reduced cardiac output reflecting reduced left heart filling.

### Cardiac tamponade

When the intrapericardial pressure exceeds right heart filling pressure (diastole), impaired filling of the chambers results in tamponade. A pericardial effusion is usually readily identified by echo, although size is no guide to the presence of tamponade. Fluid in the pericardial space is generally easy to differentiate from a pericardial fat pad or a pleural effusion. The crucial echo findings in RACE establishing the presence of tamponade and the need for rapid drainage are either right atrial wall systolic collapse for longer than one-third of the cardiac cycle, right ventricular wall diastolic collapse, and a dilated IVC [50].

Doppler interrogation across the valves by the advanced user can be used for added diagnostic support. Normal respiratory variation results in an increase of tricuspid flow during inspiration and decrease during expiration with reciprocal changes occurring with mitral valve flow. Increases in peak tricuspid velocity are usually <25 % and peak mitral velocity <15 %, whereas with tamponade the variation is much greater.

Echo is the investigation of choice in suspected cardiac tamponade, with the diagnosis generally easy to make when aligned with clinical findings. It also assists with urgent pericardiocentesis.

### Dynamic LVOT obstruction

The true incidence of dynamic left ventricular outflow obstruction in critically ill patients is unknown. It requires advanced echo Doppler expertise and in the past has usually gone unrecognized. Left ventricular wall hypertrophy classically has alerted the clinician to the possibility of hypertrophic obstructive cardiomyopathy and searching for LVOT obstruction would be seen as standard practice.

Dynamic LVOT obstruction can be present in the aged ambulatory population in the absence of wall hypertrophy [51]. Factors that make the critically ill population more susceptible, apart from age, include tachycardia, hypovolemia, and inotropes [52, 53]. 2D echo examination reveals close approximation of lateral wall and septum, plus systolic anterior motion of the anterior mitral leaflet. TEE examination often supplements the TTE approach. Color Doppler will reveal turbulent flow through the LVOT with continuous wave Doppler picking up high velocities indicating obstructive and subsequent pulsed-wave Doppler identifying exactly where that obstruction occurs. A classic spectral Doppler pattern is the so‐called “dagger” shape LVOT flow. Treatment includes re‐establishing an adequate intravascular volume, reducing heart rate to enhance diastolic filling time, and ceasing inotropes (Fig. 4).

## Septic shock

A variety of cardiac changes can be associated with septic shock, although a normal study also is not unusual (Table 2). Abnormalities in left ventricular systolic function, left ventricular diastolic function, and right ventricular function have all been described [54]. Contractile impairment may be exhibited as specific patterns such as seen in Takutsubo syndrome with apical akinesis and ballooning accompanied by good basal left ventricular contraction. Occasionally, LVOT obstruction is also described [55].

**Table 2:** Cardiac abnormalities in severe sepsis

Left ventricular dilatation
Left ventricular contraction impairment
Global
Segmental
Left ventricular diastolic dysfunction
Right ventricle systolic/diastolic dysfunction
Ventricular outflow obstruction
Valvular lesions
Functional
Endocarditis

A variety of patterns can occur in septic cardiomyopathy, including global left and/or right ventricular hypokinesis, left ventricular segmental wall motion defect patterns, and subtle changes only identified on sensitive examination, such as with speckle tracking using global longitudinal strain [56]. Importantly, the contractile dysfunction is almost always reversible over days, unless concomitant underlying coronary artery disease or myocarditis are present. Measurement of ventricular preload using echo to optimize a fluid management strategy is recommended. A major pathological contribution to shock in sepsis is peripheral vasoplegia and although this is not measurable with echo, the cardiac findings can be taken into account when estimating it. For example, in shock a hyperdynamic, well filled left ventricle is usually a clue to the presence of marked peripheral vasodilatation. Echo has a pertinent role in evaluating the valves in septic shock, both structurally and functionally. Endocarditis or peri‐valvular abscesses may be the cause of shock. TEE is the preferred technique, although TTE can still be valuable in the acute setting. The severity of any valve functional abnormality needs to be assessed and more expert examination sought where necessary, especially where prosthetic valves or congenital heart disease exist.

## Other causes of shock

Anaphylactic, neurogenic, hypo‐adrenalism, and other less common causes of shock will be assisted by the application of urgent echocardiography, sometimes in directing the clinician away from the heart as a cause of shock in the presence of a normal study.

## Conclusions

Echocardiography is perhaps the most single useful tool in the diagnosis and management of shock, particularly where the etiology is undifferentiated or multifactorial. Non‐invasive and rapid to initiate, it can be applied at the bedside anytime during the day or night. An initial basic or RACE study can lead to commencement of treatment, with a more advanced study subsequently providing incremental and vital additional information.

## Abbreviations

2D: Two-dimensional
AUC: Area under curve
CO: Cardiac output
CSA: Cross-sectional area
ICU: Intensive care unit
IVC: Inferior vena cava
LAP: Left atrial pressure
LVEF: Left ventricular ejection fraction
LVOT: Left ventricular output tract
PAcT: Pulmonary acceleration time
PLR: Passive leg raising
RACE: Rapid assessment by cardiac echo
RAP: Right atrial pressure
SV: Stroke volume
TDI: Tissue Doppler imaging
TEE: Transesphageal echocardiogram
TTE: Transthoracic echocardiogram
VTI: Velocity time integral
